# The adjuvanted recombinant zoster vaccine is efficacious and safe in Asian adults ≥ 50 years of age: a sub-cohort analysis of the ZOE-50 and ZOE-70 randomized trials

**DOI:** 10.1080/21645515.2020.1859321

**Published:** 2021-02-19

**Authors:** Joon Hyung Kim, John Diaz-Decaro, Ning Jiang, Shinn-Jang Hwang, Eun Ju Choo, Maribel Co, Andrew Hastie, David Shu Cheong Hui, Junya Irimajiri, Jacob Lee, Edward Man-Fuk Leung, Haiwen Tang, Tomomi Tsuru, Philip Watson, Zhenhua Wu, Chong-Jen Yu, Yanfei Yuan, Toufik Zahaf, Anthony L. Cunningham, Anne Schuind

**Affiliations:** a GSK, Rockville, MD, USA; b GSK, Beijing, China; c Department of Family Medicine, Taipei Veterans General Hospital, Taipei City, Taiwan; d National Yang Ming University School of Medicine, Taipei City, Taiwan; e Department of Infectious Diseases, SoonChunhyang University Bucheon Hospital, Bucheon-si, Republic of Korea; f GSK, Wavre, Belgium; g Department of Medicine and Therapeutics, The Chinese University of Hong Kong Prince of Wales Hospital, Sha Tin, Hong Kong; h Department of Dermatology, Shonan Kamakura General Hospital, Kanagawa, Japan; i Department of Internal Medicine, Division of Infectious Diseases, Kangnam Sacred Heart Hospital, Seoul, Republic of Korea; j Department of Medicine and Geriatrics, Division of Geriatric Medicine and Rehabilitation, United Christian Hospital, Kwun Tong, Hong Kong; k GSK, Shanghai, China; l Medical CO.LTA PS Clinic, Fukuoka, Japan; m GSK, Middlesex, UK; n Department of Internal Medicine, National Taiwan University Hospital, Taipei City, Taiwan; o National Taiwan University Hospital Biomedical Park Branch Hospital, Zhubei City, Hsinchu County, Taiwan; p The Westmead Institute for Medical Research, Westmead, Australia; q University of Sydney, Sydney, Australia

**Keywords:** Adjuvanted recombinant zoster vaccine, herpes zoster, postherpetic neuralgia, efficacy, safety, Asian population

## Abstract

In two large clinical trials (ZOE-50 [NCT01165177] and ZOE-70 [NCT01165229]), two doses of the adjuvanted recombinant zoster vaccine (RZV) demonstrated >90% efficacy (VE) against herpes zoster (HZ) in adults ≥50 years of age (YOA). This post-hoc analysis assessed the VE against HZ and postherpetic neuralgia (PHN), in participants from Asian study sites enrolled in ZOE-50/70. Reactogenicity and safety were also assessed. Participants ≥50 YOA were randomized 1:1 to receive 2 doses of either RZV or placebo, 2 months apart. VE was evaluated for a median follow-up of 4 years post-vaccination overall and by age in the ZOE-50 Asian population ≥50 YOA and in the pooled ZOE-50/70 Asian population ≥70 YOA. Of the 2,729 participants included in the ZOE-50 Asian population ≥50 YOA, 3 RZV and 66 placebo recipients reported a confirmed HZ episode. Overall VE was 95.6% (95% confidence interval [CI]: 86.4–99.1) against HZ and 100% (95% CI: 35.44–100) against PHN. In the pooled ZOE-50/70 Asian population ≥70 YOA, 4 RZV and 75 placebo recipients out of the 2,723 participants reported a confirmed HZ episode. Overall VE was 94.7% (95% CI: 85.9–98.6) against HZ and 89.8% (95% CI: 28.39–99.77) against PHN. Pain and myalgia were the most frequent solicited local and general adverse events, respectively, in both populations. No safety concern was identified during the study periods. RZV is highly efficacious against HZ and PHN and has an acceptable safety profile in Asian populations ≥50 YOA, similar to what was observed in the general ZOE-50/70 populations.

**Trademark statement**: *Shingrix* is a trademark owned by or licensed to the GSK group of companies.

## Introduction

After primary infection causing varicella (chickenpox), the varicella-zoster virus (VZV) establishes latency in the sensory ganglia and may later reactivate to cause herpes zoster (HZ), also known as shingles. HZ, typically characterized by a painful, dermatomal rash, causes a significant global health burden^1–3^ and is associated with substantial morbidity and impact on quality of life among older adults, in whom most cases occur.^4–6^ Immunocompromised individuals are also at increased risk of developing HZ.^7,8^ The most frequent complication of HZ is postherpetic neuralgia (PHN), occurring in up to 30% of patients.^2,9,10^ PHN substantially impacts physical, psychological, and social functioning of affected individuals.^4^ The incidence of HZ increases with age and in the presence of immunocompromising conditions, both of which lead to declines in cell-mediated immunity.^3^ In a recent systematic review, female sex, ethnicity, genetic predisposition, and comorbidities were also associated with increased risk of HZ.^11^

The adjuvanted recombinant zoster vaccine (RZV; *Shingrix*; GSK) consists of a recombinant VZV glycoprotein E (gE) and the liposome-based AS01_B_ adjuvant system (containing 50 μg of 3-*O*-desacyl-4ʹ-monophosphoryl lipid A and 50 μg of *Quillaja saponaria* Molina, fraction 21 [licensed by GSK from Antigenics LLC, a wholly owned subsidiary of Agenus Inc., a Delaware, USA corporation] and liposome). The resulting formulation enhances cell-mediated immune responses and overcomes immunosenescence in both healthy and immunocompromised adult populations.^12^ The efficacy and safety of RZV in older adults were evaluated in two large pivotal Phase III randomized, observer-blind, placebo-controlled trials comprising approximately 30,000 adults ≥50 years of age (YOA) (ZOE-50, NCT01165177) and ≥70 YOA (ZOE-70, NCT01165229) across 18 countries in North America, Latin America, Europe, Asia, and Australia.^13,14^ Over a median follow-up of 4 years in adults ≥50 and ≥70 YOA, RZV demonstrated 97.2% and 91.3% efficacy against HZ, and 91.2% and 88.8% efficacy against PHN, respectively.^13,14^ The vaccine appeared efficacious against HZ and PHN irrespective of sex, region, or geographic ancestry/ethnicity.^15^ In several countries worldwide, RZV is currently licensed and recommended for the prevention of HZ and, in some cases, PHN in adults ≥50 YOA.^16,17^ In Asia, RZV was approved in Japan (2018) and China (2019) for the prevention of HZ in adults ≥50 YOA.^18,19^ Licensure in other Asian countries is also anticipated.

**Figure 1.**
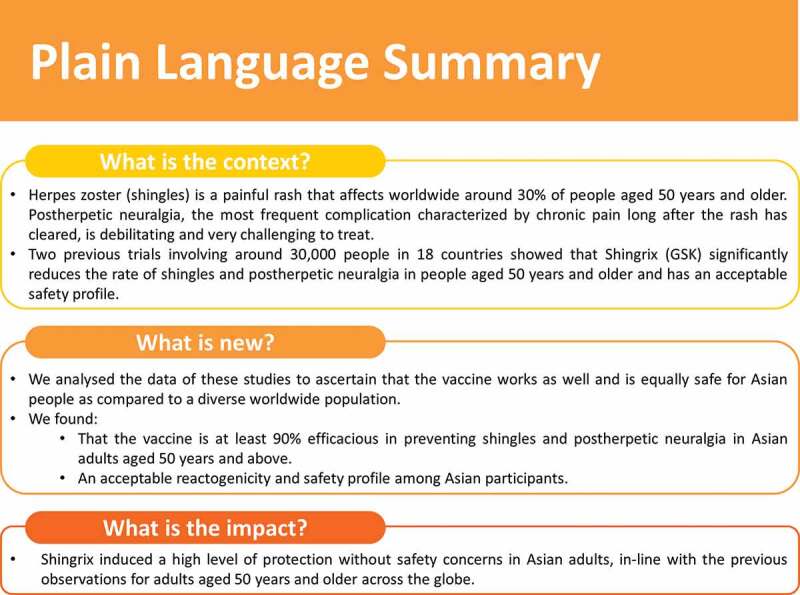
Plain language summary

In the Asia-Pacific region, limited data are available on HZ incidence, although there appears to be no apparent difference in HZ epidemiology nor risk factors for developing HZ between this region and other parts of the world.^2,20–23^ As HZ-specific vaccine-related data is lacking in Asia, there is a large interest for information on HZ vaccine safety, efficacy, and effectiveness in the local populations.^24^ Therefore, we considered it important to perform this post-hoc analysis of the pivotal ZOE-50 and ZOE-70 trials, focusing on the Asian participants. We report the efficacy of RZV against HZ and its major complication, PHN in the Asian sub-cohort including adults from Hong Kong, Japan, South Korea, and Taiwan. Reactogenicity and safety were also assessed. A plain language summary contextualizing the results and potential clinical research relevance and impact is presented in Figure 1.

## Methods

### Study design and participants

The ZOE-50/70 (Zoster-006/-022) clinical trials had a similar design and were conducted in parallel at the same centers, using methodologies that allowed the pooling of data. Protocol summaries are available at http://www.gsk-clinicalstudyregister.com (studies 110390 and 113077). Anonymized individual participant data and study documents can be requested for further research at www.clinicalstudydatarequest.com. Adults aged ≥50 years were eligible for participation unless they had a history of HZ, had previously been vaccinated against varicella or HZ, or had an immunosuppressive condition. Detailed inclusion and exclusion criteria are presented in the **Supplementary material**. Participants were randomized 1:1 to receive 2 doses of either RZV or saline placebo 2 months apart. Participants ≥70 YOA were first randomly allocated to either the ZOE-50 or ZOE-70 study and then randomized to receive either vaccine or placebo. Both studies were conducted in compliance with the Declaration of Helsinki, the principles of Good Clinical Practice, and all applicable regulatory requirements. Study protocols were approved by the appropriate independent ethics committee or institutional review board at each study center. Written informed consent was obtained from all participants before entering the study.

### Assessment of vaccine efficacy (VE)

VE against HZ and PHN was evaluated in adults ≥50 YOA included in the modified total vaccinated cohort (mTVC) of the ZOE-50 study (referred to as ZOE-50 Asian population ≥50 YOA hereafter) and in adults ≥70 YOA from the ZOE-50/70 trials included in the pooled mTVC (pooled ZOE-50/70 Asian population ≥70 YOA). The mTVC for both populations included participants from the ZOE-50 and/or ZOE-70 total vaccinated cohort [TVC] who received both doses and had no confirmed HZ episode within 30 days post-dose 2.

A suspected HZ case, defined as a new unilateral rash accompanied by pain and no alternative diagnosis, was confirmed from lesion samples by polymerase chain reaction (PCR). In the case that the PCR result was considered inconclusive or if samples were not available, final diagnosis was determined by an HZ ascertainment committee for case confirmation.^13,14^ PHN was defined as severe pain that persisted or developed more than 90 days after rash onset. For efficacy assessment, only the first confirmed HZ episode was considered, and the follow-up period for the efficacy assessment ended at the time of the first occurrence, if the participant was lost to follow-up, or at study end.

### Assessment of reactogenicity and safety

Reactogenicity was evaluated in a sub-cohort of participants that recorded solicited local (pain, redness, swelling) and general (fatigue, gastrointestinal symptoms, headache, myalgia, shivering, fever) adverse events (AEs) on diary cards for 7 days after each vaccination. Safety assessment was performed on the TVC, including all participants enrolled from Asia, who received at least one dose of RZV or placebo. Unsolicited AEs were collected for 30 days after each vaccination. If present, AEs were graded on a three-point scale from 1 (mild) to 3 (severe AE preventing normal daily activity). Serious AEs (SAEs) were collected up to 12 months post-dose 2, while SAEs with causal relationship to the vaccination, fatal SAEs, and potential immune-mediated diseases (pIMDs; new onset and possible exacerbations)^25^ were collected over the entire study periods. Causality of AEs to study vaccination was assessed by the investigator (as defined in the protocol). Results on pooled safety data from ZOE-50/70 trials have been recently published.^26^

### Statistical analysis

The pivotal studies were not designed to assess VE in study sub-populations, thus these post-hoc analyses were descriptive. VE was defined as 1 minus the ratio of HZ incidence in the RZV group over that in the Placebo group and calculated using the Poisson method. VE was evaluated for a median follow-up of 4 years post-vaccination overall and by age (50–59 YOA, 60–69 YOA, 70–79 YOA, ≥70 YOA, ≥80 YOA). The percentage of participants with at least one event was calculated with the exact 95% confidence interval (CI). All statistical analyses were performed using SAS software 9.2 (SAS Institute), and StatXact software 8.1 (Cytel).

## Results

### Study population results

In the ZOE-50 Asian population ≥50 YOA, 1,432 RZV and 1,434 placebo recipients were included in the TVC, having a mean age of 62.0 years in both groups (Table 1). Of them, 1,357 (94.8%) RZV and 1,378 (96.1%) placebo recipients received a complete 2-dose regimen; 815 and 813, respectively, were included in the diary card sub-cohort. The mTVC consisted of 1,357 RZV and 1,372 placebo recipients.

**Table 1.** Demographic characteristics of participants (total vaccinated cohort)

	ZOE-50 Asian population ≥50 YOA		Pooled ZOE-50/70 Asian population ≥70 YOA	
	RZV (N = 1432)	Placebo (N = 1434)	RZV (N = 1471)	Placebo (N = 1472)
Number of participants, n (%)				
Hong Kong	236 (16.5)	234 (16.3)	126 (8.6)	123 (8.4)
Japan	288 (20.1)	289 (20.2)	322 (21.9)	322 (21.9)
South Korea	268 (18.7)	271 (18.9)	331 (22.5)	335 (22.7)
Taiwan	640 (44.7)	640 (44.6)	692 (47.0)	692 (47.0)
Age at dose 1 (years±SD)	62.0 ± 9.1	62.0 ± 9.0	75.9 ± 4.7	76.0 ± 4.7
Female, n (%)	880 (61.5)	863 (60.2)	740 (50.3)	768 (52.2)

RZV, participants receiving the adjuvanted recombinant zoster vaccine; Placebo, participants receiving placebo; YOA, years of age; N, total number of participants; n (%), number (percentage) of participants in each category; SD, standard deviation

In the pooled ZOE-50/70 Asian population ≥70 YOA, 1,471 RZV and 1,472 placebo recipients with a mean age of 75.9 years and 76.0 years, respectively, were included in the TVC (Table 1). Of them, 1,348 (91.6%) and 1,382 (93.9%) participants, respectively, received both RZV/placebo doses and 411 and 412 participants were included in the diary card sub-cohort. The mTVC comprised of 1,347 RZV and 1,376 placebo recipients.

The most frequent reasons for withdrawal from the study were consent withdrawal (not due to an AE), SAE, and loss to follow-up in both ≥50 YOA and pooled ≥70 YOA Asian populations.

### VE against HZ

**Figure 2.**
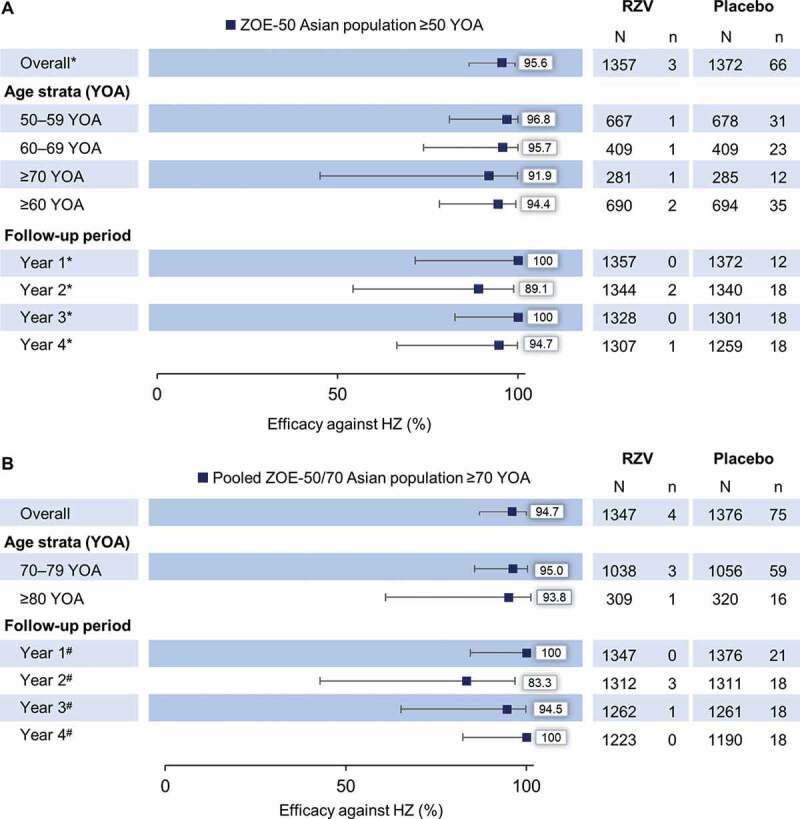
Vaccine efficacy against first or only episode of HZ by age and follow-up year in (A) ZOE-50 Asian population ≥50 YOA and (B) pooled ZOE-50/70 Asian population ≥70 YOA (modified total vaccinated cohort)

HZ, herpes zoster; RZV, participants receiving the adjuvanted recombinant zoster vaccine; Placebo, participants receiving placebo; YOA, years of age; N, number of participants; n, number of confirmed HZ episodes. *vaccine efficacy adjusted by age strata; ^#^vaccine efficacy adjusted by age strata and region. Year 1, from 30 days to 395 days post-dose 2; Year 2, from >395 days to 760 days post-dose 2; Year 3, from >760 days to 1125 days post-dose 2; Year 4, from >1125 days post-dose 2 until last contact date. Error bars depict 95% confidence intervals.

In the ZOE-50 Asian population ≥50 YOA, overall VE against HZ was 95.6% (95% CI: 86.4–99.1) (Figure 2A). Three participants reported at least one confirmed HZ episode in the RZV group; one participant in each of the 50–59 YOA, 60–69 YOA, and ≥70 YOA strata. Two HZ episodes were reported in Year 2 and one HZ episode in Year 4. In the Placebo group, a total of 66 participants reported at least one confirmed HZ episode: 31 in the 50–59 YOA, 23 in the 60–69 YOA, and 12 in the ≥70 YOA strata. Twelve episodes were reported in Year 1 and 18 episodes in each of Year 2 to 4.

In the pooled ZOE-50/70 Asian population ≥70 YOA, overall VE against HZ was 94.7% (95% CI: 85.9–98.6) (Figure 2B). Four participants (three participants in 70–79 YOA strata; one participant in ≥80 YOA strata) reported at least one HZ episode in the RZV group. Three HZ episodes were reported in Year 2 and one episode in Year 3. In the Placebo group, 75 HZ episodes were reported: 59 in the 70–79 YOA and 16 in the ≥80 YOA strata. Twenty-one episodes were reported in Year 1 and 18 episodes in each of Year 2 to 4.

### VE against complication PHN

In the ZOE-50 Asian population ≥50 YOA, no PHN episodes were reported in the RZV group, while six PHN episodes (three episodes in the 50–59 YOA, one in the 60–69 YOA, and two in the ≥70 YOA stratum) were reported in the Placebo group. VE against PHN is therefore 100% (95% CI: 35.4–100) in this population. In the pooled ZOE-50/70 Asian population ≥70 YOA, one RZV recipient (in the ≥80 YOA stratum) reported a PHN episode; in the Placebo group, a total of 10 PHN episodes (seven episodes in the 70–79 YOA and 3 episodes in the ≥80 YOA stratum) were reported, resulting an overall VE of 89.8% (95% CI: 28.4–99.8) against PHN.

### Reactogenicity

**Figure 3.**
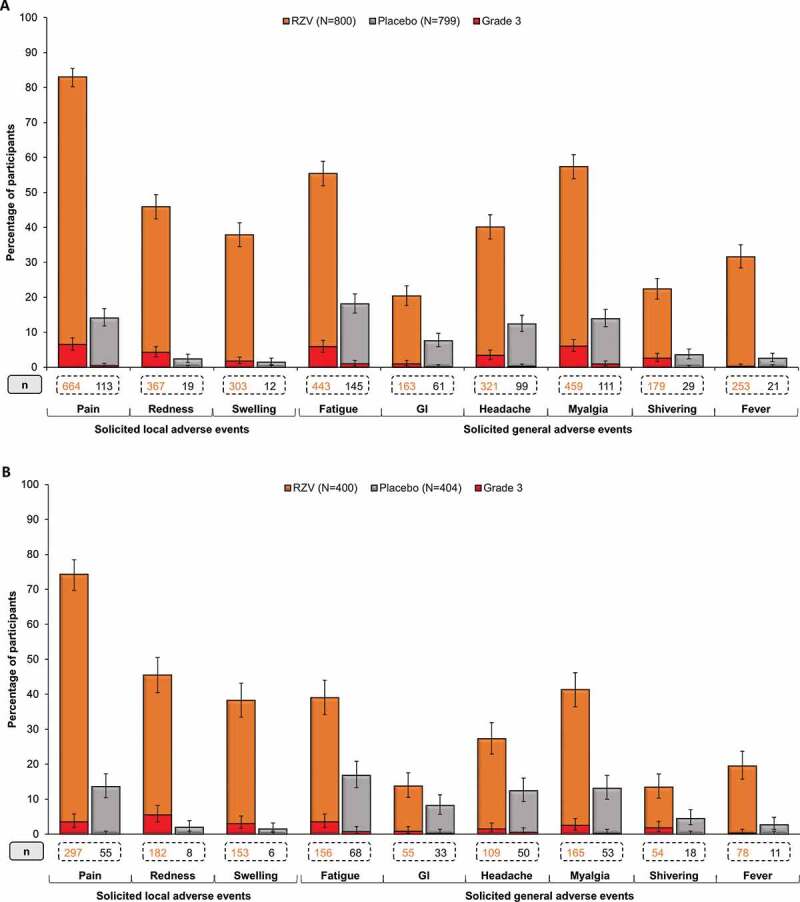
Solicited local and general adverse events (AEs) reported within 7 days after vaccination in (A) ZOE-50 Asian population ≥50 YOA and (B) pooled ZOE-50/70 Asian population ≥70 YOA (TVC diary card sub-cohort)

Reactogenicity was assessed in the diary card sub-cohort. In the ZOE-50 Asian population ≥50 YOA, 685 (85.6%) RZV, and 122 (15.3%) placebo recipients reported at least one solicited local AE, with a median duration of 3 d for each symptom; 84 (10.5%) and 3 (0.4%) participants, respectively, reported at least one grade 3 solicited local event. Pain was the most frequent solicited local AE. Grade 3 pain was reported by 52 (6.5%) RZV and 3 (0.4%) placebo recipients (Figure 3A). In the pooled ZOE-50/70 Asian population ≥70 YOA, 311 (77.8%) RZV, and 60 (14.9%) placebo recipients reported at least one solicited local AE, with a median duration of 3 d for each symptom. AEs at grade 3 intensity were reported by 38 (9.5%) RZV recipients. No grade 3 AE was recorded in the Placebo group. Pain was the most frequent solicited local AE. Fourteen (3.5%) RZV recipients reported pain at grade 3 intensity (Figure 3B).

RZV, participants receiving the adjuvanted recombinant zoster vaccine; Placebo, participants receiving placebo; TVC, total vaccinated cohort; YOA, years of age; GI, gastrointestinal symptoms; N, total number of participants; n, number of participants reporting the event. Error bars depict 95% confidence intervals.

In the ZOE-50 Asian population ≥50 YOA, 592 (74.0%) RZV, and 226 (28.3%) placebo recipients reported at least one solicited general AE, with a median duration of 2 d for most solicited general symptoms and 1 d for fever; 81 (10.1%) and 13 (1.6%) participants, respectively, reported a general AE at grade 3 intensity. The most frequently reported solicited general AEs were myalgia, fatigue, and headache in both the RZV and the Placebo group (Figure 3A). Most of the reported solicited general AEs were assessed by investigators as causally related to vaccination in both groups.

In the pooled ZOE-50/70 Asian population ≥70 YOA, at least one solicited general AE was reported by 242 (60.5%) RZV and 117 (29.0%) placebo recipients, with a median duration of 2 d for most solicited general symptoms and 1 d for fever. Twenty-two (5.5%) RZV and 4 (1.0%) placebo recipients reported a solicited general AE at grade 3 intensity. In both groups, the most common solicited general AEs were fatigue, myalgia, and headache (Figure 3B), and most of the reported general AEs were assessed as causally related to vaccination in both study groups.

### Safety

**Figure 4.**
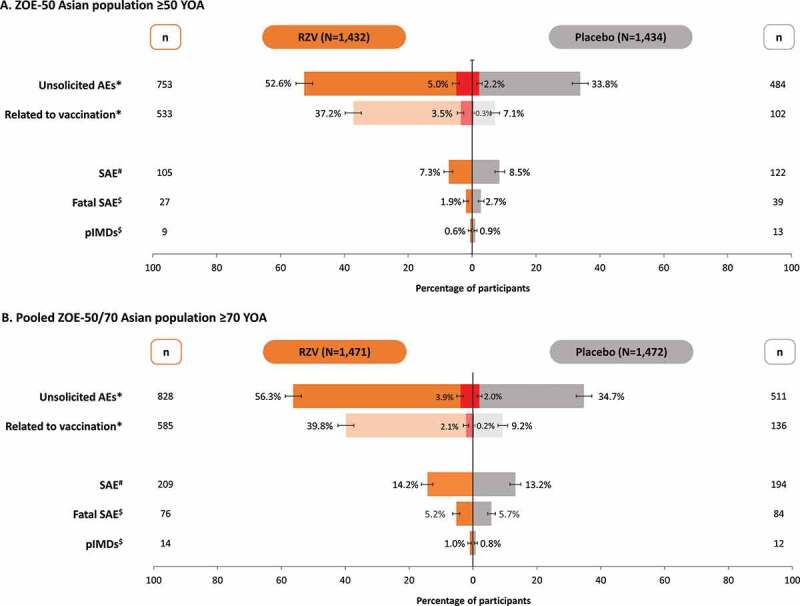
Unsolicited adverse events, serious adverse events, and potential immune-mediated diseases in (A) ZOE-50 Asian population ≥50 YOA and (B) pooled ZOE-50/70 Asian population ≥70 YOA (total vaccinated cohort)

Within the 30 days post-vaccination period in the ZOE-50 Asian population ≥50 YOA, at least one unsolicited AE was reported by 753 (52.6%) participants in the RZV and 484 (33.8%) participants in the Placebo group (Figure 4A). Grade 3 unsolicited AEs were reported by 71 (5.0%) and 31 (2.2%) participants, respectively. In the RZV group, the most common unsolicited AEs were solicited events lasting beyond the 7-day post-vaccination period (pain, swelling, erythema, and fever). In the Placebo group, the most commonly reported unsolicited AEs were nasopharyngitis, upper respiratory tract infection, headache, and cough.

RZV, participants receiving the adjuvanted recombinant zoster vaccine; Placebo, participants receiving placebo; YOA, years of age; AE, adverse events; SAE, serious adverse events; pIMD, potential immune-mediated disease; N, total number of participants; n, number of participants reporting the event. *events recorded during the 30 days post-vaccination period. ^#^Events recorded up to 12 months post-second dose. ^$^Events recorded during the entire study period. See text for additional details.

Note: Error bars represent 95% confidence interval. Red bars indicate the number of participants with grade 3 unsolicited AEs.

In the pooled ZOE-50/70 Asian population ≥70 YOA, at least one unsolicited AE was reported by 828 (56.3%) participants in the RZV and 511 (34.7%) participants in the Placebo group (Figure 4B). Grade 3 unsolicited AEs were reported by 58 (3.9%) and 29 (2.0%) participants, respectively. In the RZV group, the most common unsolicited AEs were injection site and general events (pain, swelling, erythema, and fever) reported as solicited symptoms in the diary card sub-cohort. In the Placebo group, nasopharyngitis, pain, upper respiratory tract infection, and dizziness were the most frequently reported unsolicited AEs.

In the ZOE-50 Asian population ≥50 YOA, 105 (7.3%) participants in the RZV and 122 (8.5%) participants in the Placebo group reported a SAE up to 12 months post-dose 2 (Figure 4A). None of the SAEs was assessed by the investigator as causally related to vaccination in the RZV group, while two SAEs (immune thrombocytopenic purpura and rheumatoid arthritis) were considered as related to vaccination in the Placebo group. During the entire study period, fatal SAEs were reported for 27 (1.9%) RZV and 39 (2.7%) placebo recipients. None was assessed as causally related to vaccination.

In the pooled ZOE-50/70 Asian population ≥70 YOA, SAEs were reported by 209 (14.2%) participants in the RZV group and 194 (13.2%) participants in the Placebo group up to 12 months post-dose 2. Five SAEs in the RZV group (acute myocardial infarction, arthritis bacterial, herpes zoster oticus, Guillain-Barre syndrome, and eczema) and one SAE in the Placebo group (adenocarcinoma gastric) were considered as possibly causally related to vaccination by the treating physician. Fatal SAEs were reported for 76 (5.2%) RZV and 84 (5.7%) placebo recipients. None was assessed as causally related to vaccination.

## Discussion

This post-hoc analysis focusing on Asian participants enrolled in the ZOE-50 and ZOE-70 trials demonstrated high efficacy of RZV against HZ and PHN in this population, similar to what was observed in the overall ZOE-50/70 populations.^13,14^ Point estimates for VE against HZ were >90% in all age strata in both ≥50 YOA and pooled ≥70 YOA populations. Although minor differences in reported HZ cases occurred between the follow-up years, no significant changes in VE against HZ were observed during the 4-year post-vaccination period. Only one PHN episode was reported during the study periods. Other recently published post-hoc analyses of data from the ZOE-50/70 trials demonstrated no impact of geographic ancestry/ethnicity, region, sex, or underlying medical conditions at enrollment on efficacy of RZV.^15,27,28^ VE against HZ was 97.2% (95% CI: 91.4–99.5) in Europe, 96.1% (95% CI: 88.2–99.3) in Asia/Australia, 95.7% (95% CI: 83.7–100) in North America, and 96.3% (95% CI: 77.2–100) in Latin America in the ≥50-year-olds from the ZOE-50 study and 90.1% (95% CI: 82.0–95.1) in Europe, 95.1% (95% CI: 87.0–98.7) in Asia/Australia, 90.1% (95% CI: 77.0–96.5) in North America, and 87.3% (95% CI: 58.1–97.6) in Latin America among the ≥70-year-olds from the ZOE-50/70 studies.^15^ Additionally, RZV vaccination reduced the loss of quality of life associated with both HZ and PHN and alleviated the severity of HZ-related pain in breakthrough HZ episodes.^29^

Consistent with the overall ZOE-50 and ZOE-70 study populations,^13,14^ RZV was more reactogenic than placebo in both ≥50 YOA and pooled ≥70 YOA populations in Asia. Pain was the most frequent solicited local AE, and myalgia, fatigue, and headache were the most frequent solicited general AEs. A higher percentage of participants in the RZV group reported unsolicited AEs during the 30-day post-vaccination period compared to the Placebo group. This imbalance can be attributed to AEs linked to the reactogenicity of RZV, and to the fact that only part of the Asian study population was included in the diary card sub-cohort, thus solicited to record local and general reactogenicity events on diary cards. The occurrence of SAE, fatal SAEs, and pIMDs in our Asian sub-cohort was similar between study groups, and no new concern for the safety of the vaccine was identified during the study periods, in line with the safety results on the full study populations.^26^

The overall compliance with the 2-dose regimen was 94.8% (RZV group) and 96.1% (Placebo group) in the ZOE-50 Asian population ≥50 YOA and 91.6% (RZV group) and 93.9% (Placebo group) in the pooled ZOE-50/70 Asian population ≥70 YOA. These are consistent with the dose compliance achieved in the overall populations (95.6% [RZV group] and 96.4% [Placebo group] in ZOE-50;^13^ 94.4% [RZV group] and 95.6% [Placebo group] in ZOE-70^14^) and support the favorable tolerability of RZV. A recent post-hoc analysis of the diary card sub-cohort of the pooled ZOE-50/70 population also demonstrated high compliance with the second dose (91.2%) among participants who experienced grade 3 reactogenicity event following the first RZV dose.^30^

Results of the present study need to be interpreted considering their limitations. The post-hoc nature of the analyses might be considered as a potential limitation. ZOE-50/70 studies were not statistically powered to evaluate VE in specific sub-populations. Secondly, participants included in this study might not be fully representative of the general Asian population, though the size of the sub-population was substantial, and participants were enrolled from different countries. Thirdly, this post-hoc analysis did not provide efficacy and safety data for RZV in immunocompromised individuals, as the original studies excluded individuals with any immunocompromising conditions or therapy at study entry. Nevertheless, ZOE-50/70 trials included a substantial number of frail individuals and those with comorbidities, and RZV was effective in reducing the risk of HZ in these sub-populations.^27^

## Conclusion

This sub-cohort analysis of ZOE-50/70 trials demonstrated that RZV is highly efficacious in preventing HZ and PHN up to 4 years following immunization in study participants ≥50 YOA from the Asian sub-population. VE against HZ and PHN was comparable between ≥50-year-old and ≥70-year-old Asian participants included in this post-hoc analysis and appeared similar to the VE observed in the overall ZOE-50/70 study populations. RZV showed a clinically acceptable safety profile, and no safety concerns arose in this sub-group during the study periods.

## Supplementary Material

Supplemental Material
