# Supplementary material for: The adjuvanted recombinant zoster vaccine is efficacious and safe in Asian adults ≥ 50 years of age: a sub-cohort analysis of the ZOE-50 and ZOE-70 randomized trials
Source: Hum Vaccin Immunother. 2021 Feb 19;17(7):2050–7. doi: 10.1080/21645515.2020.1859321 (PMC8189096; doi:10.1080/21645515.2020.1859321)
Supplement: Supplemental Material [file KHVI_A_1859321_SM8412.docx]

**the adjuvanted recombinant zoster vaccine is Efficacious and safe in Asian adults ≥50 years of age: a sub-cohort analysis of the ZOE-50 and ZOE-70 randomized trials**

# Supplementary material

Inclusion and exclusion criteria

Participants had to satisfy all the following criteria at study entry:

- A male or female aged 50 (ZOE-50) or 70 (ZOE-70) years or older at the time of the first vaccination;
- Written informed consent obtained from the participant;
- Participants who in the opinion of the investigator could comply with the requirements of the protocol (e.g., completion of the diary cards/questionnaires, return for follow-up visits, have regular contact to allow evaluation during the study);
- Female participants of non-childbearing potential could be enrolled in the study;
- For this study population, non-childbearing potential was defined as current tubal ligation, hysterectomy, ovariectomy or post-menopause. OR
- Female participants of childbearing potential could be enrolled in the study, if the participant had practiced adequate contraception for 30 days before vaccination, had a negative urine pregnancy test on the day of vaccination, and had agreed to continue adequate contraception during the entire treatment period and for 2 months after completion of the vaccination series.

Participants were not included in the study if any exclusion criterion applied:

- History of herpes zoster (HZ);
- Previous vaccination against varicella or HZ (either with a registered product or through participation in a previous vaccine study, and including previous vaccination with childhood varicella vaccine);
- Any confirmed or suspected immunosuppressive or immunodeficient condition resulting from disease (e.g., malignancy, HIV infection) or immunosuppressive/cytotoxic therapy (e.g., medications used during cancer chemotherapy, organ transplantation, or to treat autoimmune disorders.
- History of allergic disease or reactions likely to be exacerbated by any component of the vaccine. Additionally, allergic reactions to other material or equipment related to study participation (such as materials that may possibly contain latex -gloves, syringes, etc.) were also taken into account. The vaccine and vials in this study did not contain latex;
- Significant underlying illness that in the opinion of the investigator was expected to prevent completion of the study (e.g., life-threatening disease likely to limit survival to less than 4 years);
- Concurrently participating in another clinical study, at any time during the study period, in which the participant had been or was planned to be exposed to an investigational or a non-investigational product (pharmaceutical product or device);
- Use of any investigational or non-registered product (drug or vaccine) other than the study vaccine within 30 days preceding the first dose of study vaccine, or planned use during the study period;
- Receipt of immunoglobulins or any blood products within the 90 days preceding the first dose of study vaccine or planned administration during the study period;
- Administration or planned administration of any other immunizations within 30 days before the first or second study vaccination or scheduled within 30 days after study vaccination. However, licensed non-replicating vaccines (i.e., inactivated and subunit vaccines, including inactivated and subunit influenza vaccines for seasonal or pandemic flu, with or without adjuvant) were allowed to be administered up to 8 days before each dose or at least 14 days after any dose of study vaccine;
- Any other condition (e.g., extensive psoriasis, chronic pain syndrome, cognitive impairment, severe hearing loss) that, in the opinion of the investigator, might have interfered with the evaluations required by the study;
- Acute disease or fever at the time of enrolment; fever was defined as temperature ≥37.5°C on oral, axillary, or tympanic setting, or 38.0°C on rectal setting. The preferred route for recording temperature in this study was oral.
- Participants with a minor illness (such as mild diarrhea, mild upper respiratory infection) without fever could be enrolled at the discretion of the investigator.
- Chronic administration (defined as >15 consecutive days) of immunosuppressants or other immune-modifying drugs within six months prior to the first vaccine dose. For corticosteroids, this meant prednisone <20 mg/day, or equivalent, was allowed. Inhaled and topical steroids were allowed.
- Pregnant or lactating female;
- Female planning to become pregnant or planning to discontinue contraceptive precautions (if of childbearing potential).
